# Supplementary material for: Dynamin1 long- and short-tail isoforms exploit distinct recruitment and spatial patterns to form endocytic nanoclusters
Source: Nat Commun. 2024 May 14;15:4060. doi: 10.1038/s41467-024-47677-8 (PMC11094030; doi:10.1038/s41467-024-47677-8)
Supplement: Supplementary file 1 — Supplementary Information [file 41467_2024_47677_MOESM1_ESM.pdf]

Supplementary Information

Supplementary Figure 1

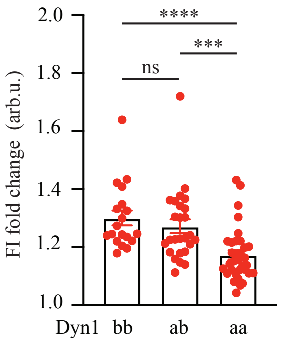

Supplementary Figure 1: Average FI fold change of the entire plasma membrane of different Dyn1 isoforms

Average FI fold change of the entire plasma membrane of PC12 cell transfected with either Dyn1bb-GFP, Dyn1ab-GFP, or Dyn1aa-GFP following Ba^{2+} stimulation. (n=19 cells for Dyn1bb, n=26 cells for Dyn1ab, n=33 cells for Dyn1aa from 4 independent experiments; non-normally distributed paired data was analysed with Wilcoxon test; ns non-significant *P*>0.05, **P*<0.05, ****P*<0.001, *****P*<0.0001; Mean ± SEM are plotted).

Supplementary Figure 2

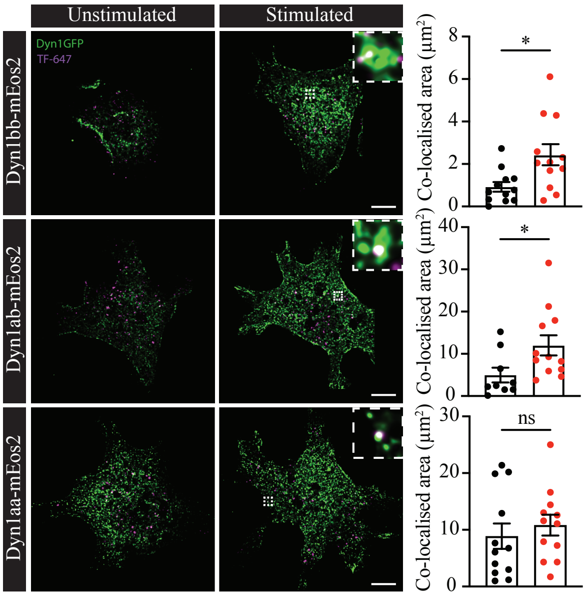

Supplementary Figure 2: Dyn1 long- and short-tail isoforms co-localize with transferrin

Dyn1bb-GFP, or Dyn1ab-GFP, or Dyn1aa-GFP transfected PC12 cells were incubated with 10µg/mL Tf-647 for 5 min in unstimulated control or Ba^{2+} stimulated (applied at 3 min) conditions, and fixed at 5 min. Example photomicrographs of PC12 cells expressing Dyn1bb-GFP, or Dyn1ab-GFP, or Dyn1aa-GFP (green) incubated with Tf-647 for 5 minutes (magenta) are show. Scale bar = 5µm. Cells were either unstimulated or stimulated with Ba^{2+} for 2 min. Dashed boxes contain zoom-in showing co-localisation of Dyn1 and Tf following stimulation. Co-localisation area (μm^2) of Dyn1bb and Tf, or Dyn1ab and Tf are significantly increased following stimulation compared to unstimulated controls (* $p<0.05$; n=12 cells). Stimulation does not alter the colocalization of Dyn1aa and Tf (ns non-significant $p>0.05$; n=12 cells).

Supplementary Figure 3

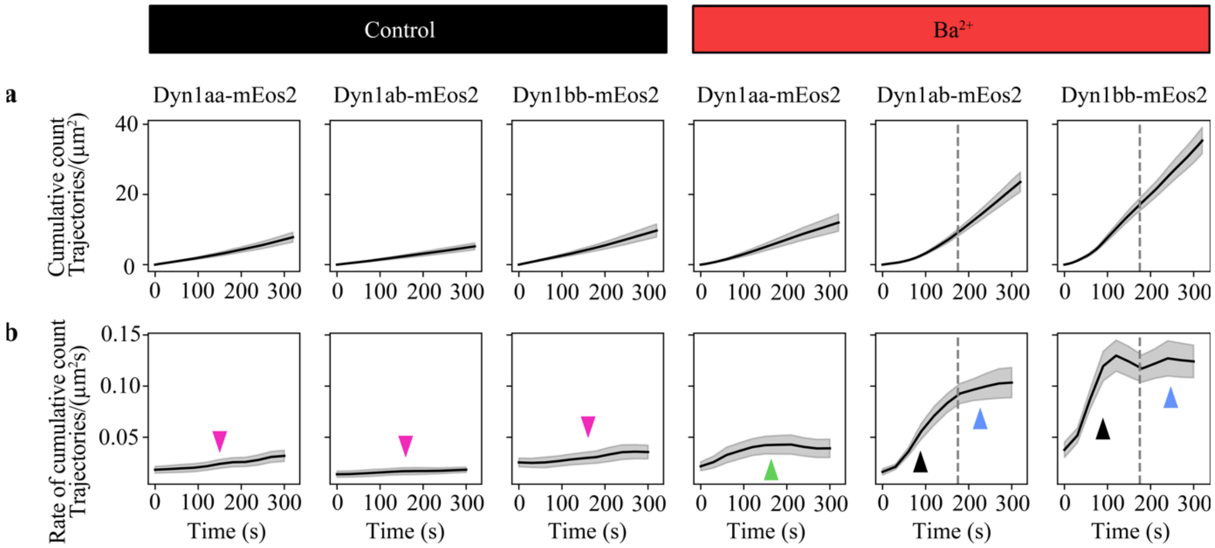

Supplementary Figure 3: Cumulative counts of Dyn1aa, Dyn1ab and Dyn1bb single-molecule trajectories

(a) Cumulative counts of single-molecule trajectories throughout acquisition and (b) rate of cumulative counts of single-molecule trajectories throughout acquisition, under control and following Ba^{2+} secretagogue stimulation for respective Dyn1 isoforms (pink, black, and blue arrowheads represent supposed steady-state, early-phase recruitment, and clustering phase, respectively; grey dotted lines at t = 175s are times when low FI area peaked in intensity from Fig. 1).

Supplementary Figure 4

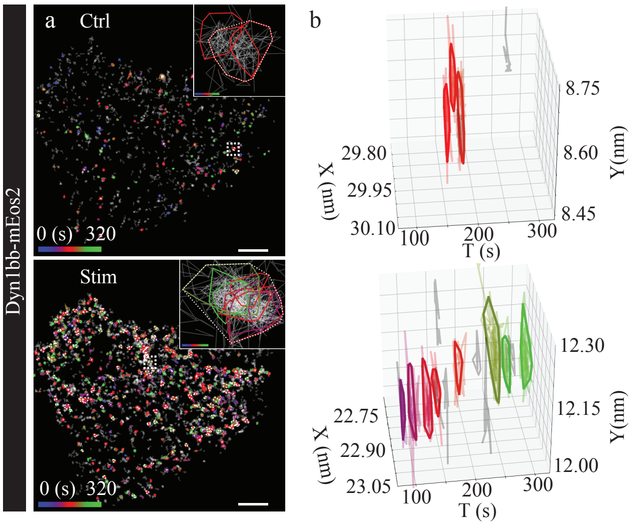

Supplementary Figure 4: Representative 2D and 3D plots of Dyn1bb-mEos2 nanoclusters in PC12 cells

(a) Representative nanoclusters of Dyn1bb-mEos2 in either unstimulated (Ctrl) or stimulated (2 mM Ba^{2+}) conditions in transfected PC12 cells. Scale bars, 5 µm. ROI (white box) from (a) is shown magnified in (b) which depicts a representative 3D [x,y,t] projection of a single spatial hotspot of Dyn1bb-mEos2 in unstimulated and stimulated conditions, which resolve into multiple spatiotemporal clusters indicated by colour.

Supplementary Figure 5

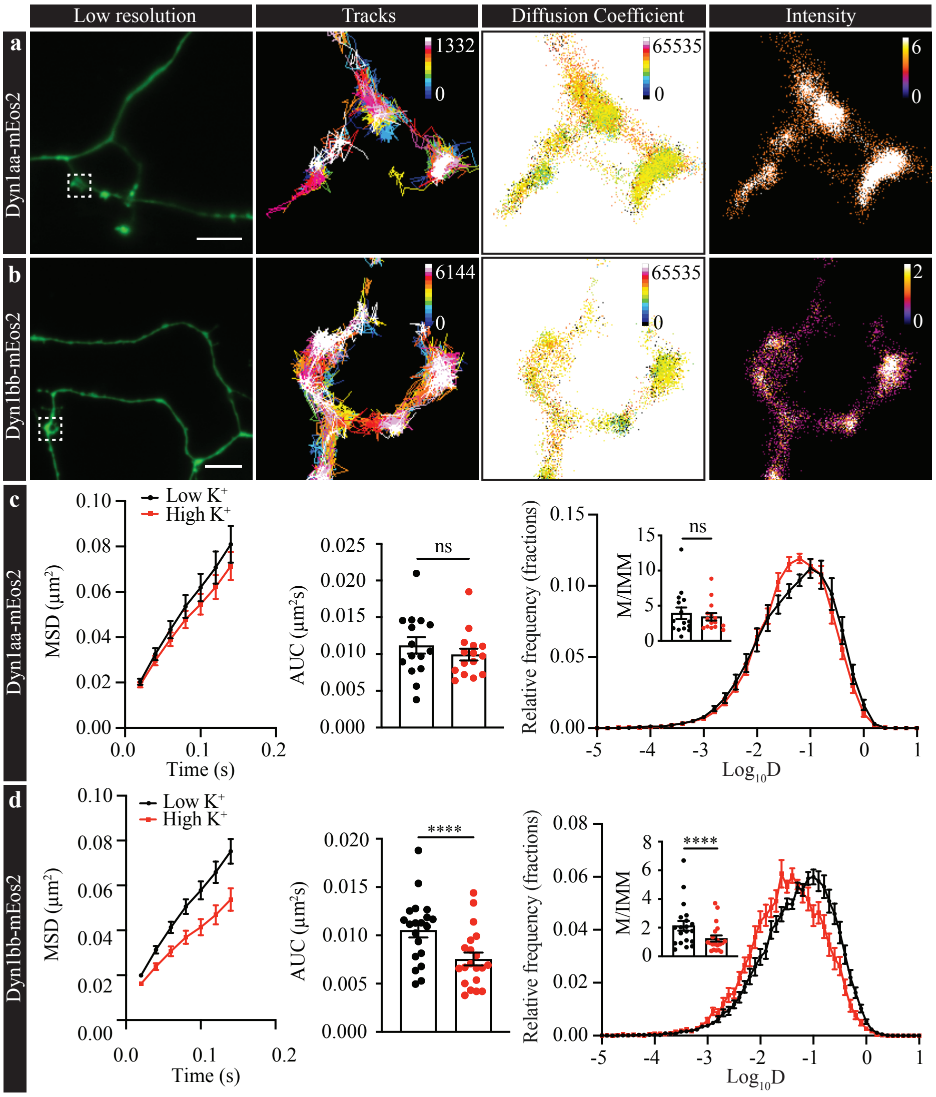

Supplementary Figure 5: Single molecule mobility dynamics of Dyn1bb and Dyn1aa in mice primary hippocampal neurons

Dyn1aa-mEos2 and Dyn1bb-mEos2 mobility was individually imaged at 50 Hz in either low K^+ (control) or high K^+ (stimulation) conditions in transfected mice primary hippocampal neurons. (a,b) Representative low-resolution images with white dotted boxes show magnified, sptPALM trajectory maps, diffusion coefficient maps and average intensity maps for (a) Dyn1aa-mEos2 and (b) Dyn1bb-mEos2 in a low K^+ condition. Scale bar, 5 µm. (c, d) The single molecule mobility in both low K^+ and high K^+ conditions was compared for (c) Dyn1aa-mEos2 and (d) Dyn1bb-mEos2, with the average MSD (µm^2) shown on the left, the AUC of the MSD (µm^{2}s) in the middle, and mean frequency distribution of $Log_{10}D$ with an M/IMM fraction insert on the right. These analyses were carried out before and after stimulation (n=20 cells for

Dyn1bb, n=15 cells for Dyn1aa from 3 independent experiments; normally distributed paired data was analysed with paired Student's *t* test; ns non-significant $P>0.05$, ****$P<0.0001$; Mean ± SEM are plotted).

Supplementary Figure 6

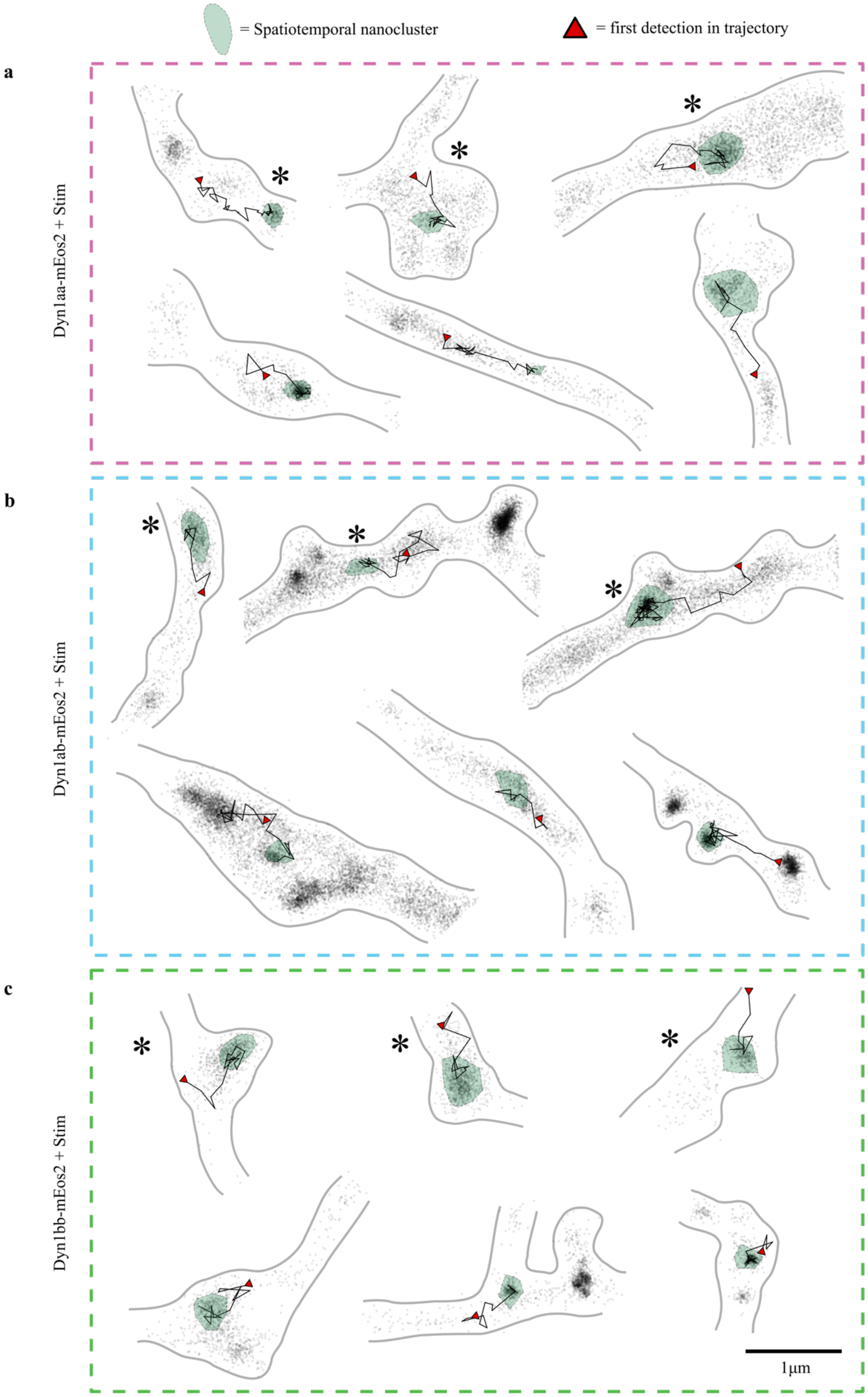

Supplementary Figure 6: Representative two-dimensional sampling followed by cluster confinement of Dyn1aa, Dyn1ab and Dyn1bb in primary hippocampal neurons

Representative trajectories are plotted alongside BOOSH-derived spatiotemporal nanocluster (green) and single-molecule detections (grey points) displayed within axon for (a) Dyn1aa-mEos2, (b) Dyn1ab-mEos2, and (c) Dyn1bb-mEos2. (*) represents those trajectories that were featured in Figure 5d.

Supplementary Figure 7

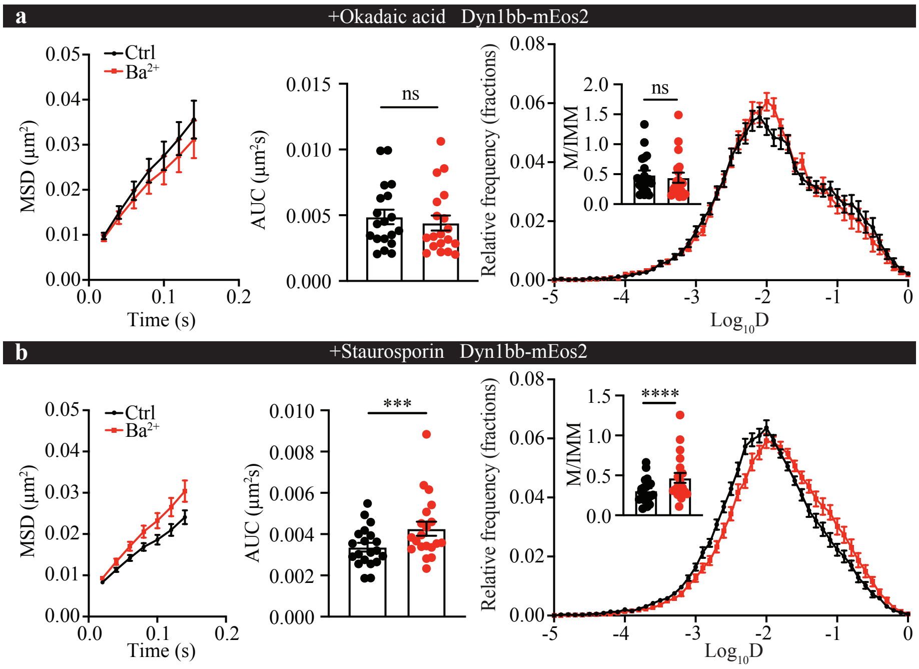

Supplementary Figure 7: Phosphorylation and dephosphorylation affect the second concentration/clustering phase of short-tail Dyn1 isoforms

PC12 cells expressing Dyn1bb-mEos2 were pre-treated with either okadaic acid (1 µM for 30 min) or staurosporine (1 µM for 30 min). and imaged at 50 Hz in either unstimulated or stimulated (2 mM Ba^{2+}) conditions. The single molecule mobility of Dyn1bb-mEos2 in unstimulated and stimulated PC12 cells was compared in the (a) okadaic acid condition and (b) staurosporine condition. From left to right: the average MSD (µm^2), AUC of the MSD (arbitrary units; a.u), and mean frequency distribution of $Log_{10}D$ with an insert showing the M/IMM fraction. (n=19 cells for the okadaic acid condition, n=20 for the staurosporine condition from 3 independent experiments; non-normally distributed paired data was analysed

with the Wilcoxon test; ns non-significant $P>0.05$, ***$P<0.001$, ****$P<0.0001$; Mean ± SEM are plotted).

Supplementary Figure 8

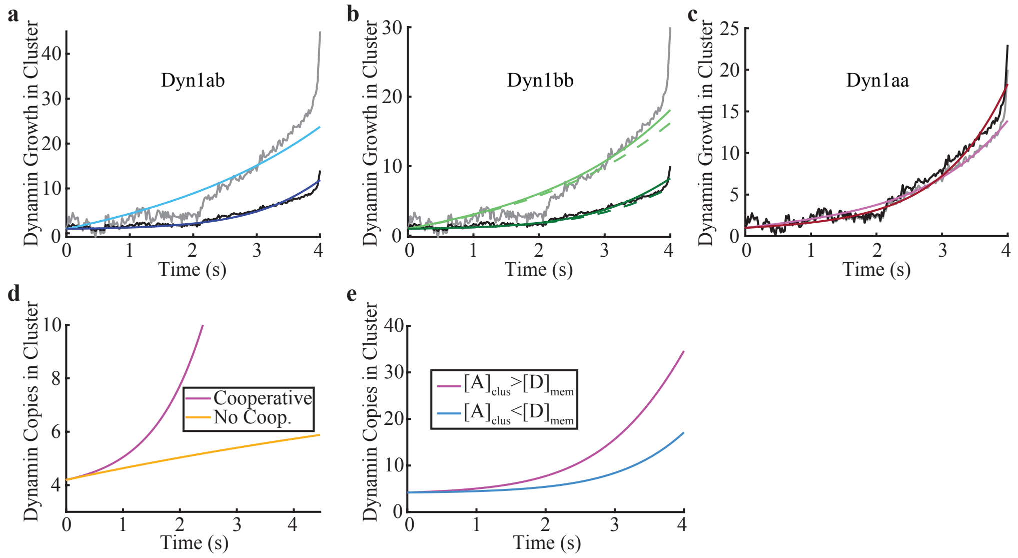

Supplementary Figure 8: Model reproduces dynamics of dynamin accumulation in clusters for different isoforms

(a-c). Experimental curves are shown in grey and black for all figures. The scaling on the y-axis is controlled by the initial copies found in the model, and the relative increase is constrained by isoform-specific measurements (see Table 2). The initial copies of dynamin post-stimulation are coupled to the copies prior: they are increased by the factor of dynamin density increased on the membrane surface, as measured using SPT. Here we normalize the growth relative to the initial copies, so it starts at 1 for all curves. The factor increase for Dyn1ab is 7, for Dyn1bb is 5, and for Dyn1aa is only 2. (a) Dyn1ab pre-stimulation in light blue, and post-stimulation in dark blue. (b) Dyn1bb pre-stimulation in light green, post-stimulation in dark green. (c) Dyn1aa pre-stimulation in pink and post-stimulation in dark red. (d) The relative increase in density or copies within the cluster, has a shape that is sensitive to the addition of cooperativity. We introduce cooperativity in the model by allowing dynamin in the cluster to recruit itself via a rate k_{dyn} (pink curve). If $k_{dyn}=0$, no cooperativity occurs (gold curve), and the growth of dynamin in the cluster is linear before plateauing, which does not agree with the experimental observations. (e) The rise of dynamin in the cluster can be accelerated by increasing the density of 'activators', or molecules that are restricted to the

cluster and recruit dynamin. These molecules represent dynamin binding partners that assemble into clusters prior to dynamin, such as adaptor proteins.

Supplementary Figure 9

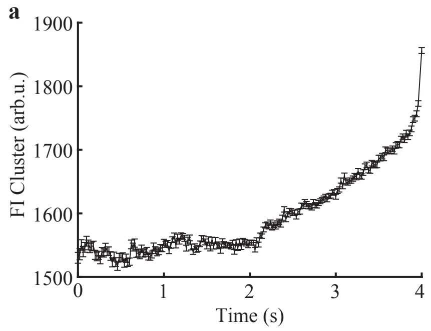

Supplementary Figure 9. Fluorescence intensity measured during cluster formation

Because the extent of background fluorescence is not known from these measurements, we use the kinetics of this experimental data, but we scale the relative increase in density from time zero to time 4 s by the density increases during cluster formation measured using single-particle tracking (SPT). The density increases measured using SPT (Table 2) indicate an increase in dynamin copy numbers in clusters that seems much more consistent with formation of dynamin assemblies (10-30x increase). Our model does not describe the subsequent decrease in dynamin density, presumably following fission events, as we do not include a mechanism to stop assembly (such as fission).

Supplementary Table 1. Nanoclustering metrics of DynNB-mEos2 in various cellular settings

	Cluster radius (µm)	Cluster density (clusters/µm^2)	Cluster lifetime (s)	Clusters in hotspots (%)
MEF_DynNB-mEos2	0.085 ± 0.003	0.03 ± 0.008	5.87 ± 0.47	3.3 ± 0.97
MEF_TMX_Dyn1bb-mEos2_serum depletion	0.071 ± 0.002	0.08 ± 0.01	5.13 ± 0.32	0.79 ± 0.25
MEF_TMX_Dyn1bb-mEos2_serum	0.068 ± 0.002	0.19 ± 0.06	6.00 ± 0.17	1.27 ± 0.35
PC12_DynNB-mEos2_Ctrl	0.075 ± 0.002	0.18 ± 0.07	10.93 ± 1.89	10.17 ± 4.73
PC12_DynNB-mEos2_Stim	0.080 ± 0.004	0.21 ± 0.09	10.95 ± 1.36	4.33 ± 1.58

Supplementary Table 2. Parameters derived from sptPALM analysis

	Dyn1bb	Dyn1ab	Dyn1aa
Factor Density increase in clusters, Pre-stim (via SPT)	30.7	44.8	19.7
Factor Density increase in clusters, Post-stim (via SPT)	10.4	14.0	22.8
Factor membrane-bound density increase following stim (via SPT)	5.2	7.0	2.1
Occupied area/cluster, Pre-stim (μm^2/cluster)	1.85	4.34	2.85
Occupied area/cluster, Post-stim (μm^2/cluster)	0.32	0.57	1.43
Cluster Radius, Pre-stim (μm)	0.084	0.086	0.080
Cluster Radius, Post-stim (μm)	0.096	0.091	0.081

Diffusion Constant Pre-Stim ($\mu m^2/s$)	0.045	0.06	0.03
Diffusion Constant Post-Stim ($\mu m^2/s$)	0.026	0.04	0.03

Supplementary Table 3. Parameters to optimize and allowed ranges

Optimizable parameter	Accepted values
$[D]_{mem}$ ($1/\mu m^2$)	10-80
$[D]_{sol}$ (μM)	0.1-10
$[R]_{mem}$ ($1/\mu m^2$)	1-500
$[A]_{clus}$ ($1/\mu m^2$)	$[D]_{mem} x$[0.01-100]
k_{frev} ($\mu M^{-1} s^{-1}$)	10^{-4}-1
k_b (s^{-1})	Constrain to equilibrium
$k_{mem,pre}$ ($\mu M^{-1} s^{-1}$)	10^{-4}-1
$k_{mem,post}$ ($\mu M^{-1} s^{-1}$)	10^{-4}-1
k_{Dydy} ($\mu M^{-1} s^{-1}$)	$k_{mem,pre} x$[0.01-100]
h (μm)	0.01
V/A (μm)	1.9
