# Supplementary material for: Dynamin1 long- and short-tail isoforms exploit distinct recruitment and spatial patterns to form endocytic nanoclusters
Source: Nat Commun. 2024 May 14;15:4060. doi: 10.1038/s41467-024-47677-8 (PMC11094030; doi:10.1038/s41467-024-47677-8)
Supplement: Supplementary file 3 — Description of Additional Supplementary Files [file 41467_2024_47677_MOESM3_ESM.pdf]

Description of Additional Supplementary Files

File Name: Supplementary Movie 1
Description: Dyn1bb-GFP transfected PC12 cells imaged with TIRF microscopy. HFL and LFA as well as discrete small clusters were clearly appeared at different times after stimulation.

File Name: Supplementary Movie 2
Description: NERDSS (stochastic structure-resolved reaction diffusions) simulation of Dyn1bb recruitment to clusters pre-stimulation. Parameters in Fig. 8 legend and same geometry as Fig. 8b. Gray molecules are 'activators' localized to the clusters. Pink molecules are Dyn1bb localized to the membrane. Cyan molecules are Dyn1bb on the membrane and localized to the cluster. Each frame progresses 0.06 s, hence the significant displacements of particles. Solution dynamin is not shown for clarity.

File Name: Supplementary Movie 3
Description: NERDSS (stochastic structure-resolved reaction diffusions) simulation of Dyn1bb recruitment to clusters pre-stimulation with solution molecules shown. Same movie as Movie 2, but now we show the solution Dyn1bb present for completeness. Solution molecules are in blue.

File Name: Supplementary Movie 4
Description: NERDSS (stochastic structure-resolved reaction diffusions) simulation of Dyn1bb recruitment to clusters post-stimulation. Parameters in Fig. 8 legend and now the geometry is based on the higher cluster density illustrated in Fig. 8c. Grey molecules are 'activators' localized to the clusters. Pink molecules are Dyn1bb localized to the membrane. Cyan molecules are Dyn1bb on the membrane and localized to the cluster. Each frame progresses 0.06 s, hence the significant displacements of particles. Solution molecules not shown for clarity.
