# Supplementary material for: Dynamin1 long- and short-tail isoforms exploit distinct recruitment and spatial patterns to form endocytic nanoclusters
Source: Nat Commun. 2024 May 14;15:4060. doi: 10.1038/s41467-024-47677-8 (PMC11094030; doi:10.1038/s41467-024-47677-8)
Supplement: Supplementary file 8 — Reporting Summary [file 41467_2024_47677_MOESM8_ESM.pdf]

nature portfolio

Corresponding author(s): Frédéric A. Meunier and Merja Joensuu

Last updated by author(s): 2 April 2024

Reporting Summary

Nature Portfolio wishes to improve the reproducibility of the work that we publish. This form provides structure for consistency and transparency in reporting. For further information on Nature Portfolio policies, see our Editorial Policies and the Editorial Policy Checklist.

Statistics

For all statistical analyses, confirm that the following items are present in the figure legend, table legend, main text, or Methods section.

n/a	Confirmed	
☐	☒	The exact sample size (*n*) for each experimental group/condition, given as a discrete number and unit of measurement
☐	☒	A statement on whether measurements were taken from distinct samples or whether the same sample was measured repeatedly
☐	☒	The statistical test(s) used AND whether they are one- or two-sided<br>*Only common tests should be described solely by name; describe more complex techniques in the Methods section.*
☒	☐	A description of all covariates tested
☒	☐	A description of any assumptions or corrections, such as tests of normality and adjustment for multiple comparisons
☐	☒	A full description of the statistical parameters including central tendency (e.g. means) or other basic estimates (e.g. regression coefficient) AND variation (e.g. standard deviation) or associated estimates of uncertainty (e.g. confidence intervals)
☐	☒	For null hypothesis testing, the test statistic (e.g. *F*, *t*, *r*) with confidence intervals, effect sizes, degrees of freedom and *P* value noted<br>*Give P values as exact values whenever suitable.*
☒	☐	For Bayesian analysis, information on the choice of priors and Markov chain Monte Carlo settings
☒	☐	For hierarchical and complex designs, identification of the appropriate level for tests and full reporting of outcomes
☒	☐	Estimates of effect sizes (e.g. Cohen's *d*, Pearson's *r*), indicating how they were calculated

Our web collection on statistics for biologists contains articles on many of the points above.

Software and code

Policy information about availability of computer code

Data collection	TIRF microscopy and super resolution microscopy data (sptPALM) were acquired using Metamorph Microscopy Automation and Image analysis Software, version 7.7.8.
Data analysis	TIRF Fluorescence Intensity (FI) Analysis is done with Fiji (imageJ) (2.0.0-rc-43/1.50e; National Institutes of Health), sptPALM data is analysed with a custom-written program for Metamorph software named PALM-Tracer, nanocluster analysis is done with out novel method named data in TRXYT format were analysed using NASTIC (Nanoscale Spatio Temporal Indexing Clustering). Prism 9 (GraphPad Software 9.5.1) is used for visualisations and statistics. All NASTIC code is available via GitHub at https://github.com/tristanwallis/smlm_clustering

For manuscripts utilizing custom algorithms or software that are central to the research but not yet described in published literature, software must be made available to editors and reviewers. We strongly encourage code deposition in a community repository (e.g. GitHub). See the Nature Portfolio guidelines for submitting code & software for further information.

Data

Policy information about availability of data

All manuscripts must include a data availability statement. This statement should provide the following information, where applicable:

- Accession codes, unique identifiers, or web links for publicly available datasets
- A description of any restrictions on data availability
- For clinical datasets or third party data, please ensure that the statement adheres to our policy

Source data is integrated into an excel file for submission.

Raw data generated in this study are available for downloading from the publicly accessible institutional data repository of The University of Queensland ((UQ eSpace): DOI: 10.48610/5d9371d.
The code repository is available at https://github.com/tristanwallis/smlm_clustering
Additional data requests can be made to the corresponding authors.

Research involving human participants, their data, or biological material

Policy information about studies with human participants or human data. See also policy information about sex, gender (identity/presentation), and sexual orientation and race, ethnicity and racism.

Reporting on sex and gender	N/A
Reporting on race, ethnicity, or other socially relevant groupings	N/A
Population characteristics	N/A
Recruitment	N/A
Ethics oversight	N/A

Note that full information on the approval of the study protocol must also be provided in the manuscript.

Field-specific reporting

Please select the one below that is the best fit for your research. If you are not sure, read the appropriate sections before making your selection.

[x] Life sciences [ ] Behavioural & social sciences [ ] Ecological, evolutionary & environmental sciences

For a reference copy of the document with all sections, see nature.com/documents/nr-reporting-summary-flat.pdf

Life sciences study design

All studies must disclose on these points even when the disclosure is negative.

Sample size	For comparison of fluorescence intensity, single molecule mobility changes and clustering metrics between unstimulated and stimulated PC12 cells, MEF cells, and hippocampal neurons, regions of interest were selected from multiple cells of each condition. Based on previous published work in our laboratory (Bademosi et al. 2016 Nature Communications and Bademosi et al. 2018 Cell Reports and Gormal et al. 2020 PNAS), we used numbers sufficient to derive statistically significant differences of the protein being studied, detail of each experiment is indicated in the figure legends. No statistical calculations were used to predict this sample size.
Data exclusions	Raw microscopy data was excluded if it exhibited drift during acquisition, which would have altered the accuracy /precision of localization. Data was excluded after analysis if it failed to yield more than a defined number of trajectories, benchmarked for each protein of interest and expected yields. We excluded cells whose acquisitions were of extremely high density (resulting in greater than 50 trajectories/um"2). This is typically done in our laboratory as very high density super resolution data leads to spurious clustering artifacts.
Replication	The experiments have all been replicated as indicated in figure legends. Variability between each preparation was minimized as each experiment was performed identically. Same cell culturing protocols, same transfection paradigm, same microscope, identical imaging parameters were used for each replication of experiments.The TIRF low resolution microscopy and super resolution microscopy acquisition parameters, and subsequent NASTIC analysis parameters are clearly defined for all figures and data used in the study.
Randomization	Morphologically similar cells with moderate level of expression were selected for TIRF microscopy fluorescence intensity assay and super resolution microscopy. All acquisitions were performed with identical microscope and imaging software settings and analysed in batches that used the same analytical parameters.
Blinding	Since comparative biological comparisons (i.e drug treatments) are not a feature of our study, blinding was not required.

Reporting for specific materials, systems and methods

We require information from authors about some types of materials, experimental systems and methods used in many studies. Here, indicate whether each material, system or method listed is relevant to your study. If you are not sure if a list item applies to your research, read the appropriate section before selecting a response.

Materials & experimental systems

n/a	Involved in the study	
☐	☒	Antibodies
☐	☒	Eukaryotic cell lines
☒	☐	Palaeontology and archaeology
☒	☐	Animals and other organisms
☒	☐	Clinical data
☒	☐	Dual use research of concern
☒	☐	Plants

Methods

n/a	Involved in the study	
☒	☐	ChIP-seq
☒	☐	Flow cytometry
☒	☐	MRI-based neuroimaging

Antibodies

Antibodies used	No immunoglobulin antibodies were used in this study. However, a fluorescently labelled nanobody were utilized to detect endogenous dyanamin: pEGFP-N1-DynNB was a generous gift from Aurélien Roux (Galli, V. et al. Elife).
Validation	A previous published nanobody DynNB targeted endogenous dynamin (Galli, V. et al. 2017, Elife) was used in this study. Photoconvertable fluorescent protein mEos2 was laballed to DynNB for our single molecule tracking experiments.

Eukaryotic cell lines

Policy information about cell lines and Sex and Gender in Research

Cell line source(s)	American Type Culture Collection (ATCC) Source PC12 cells - Rattus norvegicus, Pheochomocytoma cell line. Dyn1,2 double knockout Mouse embryonic fibroblast cell line (Ferguson, S. et al. 2009 Developmental cell) was a gift from Giuseppe Balistreri.
Authentication	Cell lines are scrutinized for morphological characteristics of their line. The cell line has not been verified genetically.
Mycoplasma contamination	All cell lines are regularly tested for Mycoplasma, all cell lines have been verified as Mycoplasma free.
Commonly misidentified lines (See ICLAC register)	N/A

Plants

Seed stocks	N/A
Novel plant genotypes	N/A
Authentication	N/A
